# Survey of *Plasmodium* in the golden-headed lion tamarin (*Leontopithecus chrysomelas*) living in urban Atlantic forest in Rio de Janeiro, Brazil

**DOI:** 10.1186/s12936-016-1155-3

**Published:** 2016-02-17

**Authors:** Elizabeth Helen Aitken, Marina Galvão Bueno, Luana dos Santos Ortolan, José M. Alvaréz, Alcides Pissinatti, Maria Cecília Martins Kierulff, José Luiz Catão-Dias, Sabrina Epiphanio

**Affiliations:** Departamento de Imunologia, Instituto de Ciências Biomédicas, Universidade de São Paulo, São Paulo, São Paulo Brazil; Department of Medicine, Peter Doherty Institute, University of Melbourne, Melbourne, Victoria Australia; Instituto Pri-Matas para Conservação da Biodiversidade (Pri-Matas), Rio de Janeiro, Brazil; Instituto de Desenvolvimento Sustentável Mamirauá (IDSM/MCTI), Tefé, Amazonas Brazil; Fundação Oswaldo Cruz, Programa Institucional Biodiversidade & Saúde, Rio de Janeiro, Brazil; Centro de Primatologia (CPRJ/INEA), Rio de Janeiro, Brazil; Programa de Pós-Graduação em Biodiversidade Tropical, Centro Universitário Norte do Espírito Santo, Universidade Federal do Espírito Santo, São Mateus, Brazil; Laboratório de Patologia Comparada de Animais Selvagens (LAPCOM), Departamento de Patologia, Faculdade de Medicina Veterinária e Zootecnia, Universidade de São Paulo, São Paulo, São Paulo Brazil; Departamento de Análises Clínicas e Toxicológicas, Faculdade de Ciências Farmacêuticas, Universidade de São Paulo, Av. Prof. Lineu Prestes, 580, Butantã, São Paulo 05508-000 Brazil

**Keywords:** *Plasmodium*, Malaria, *Leontopithecus chrysomelas*, Translocation

## Abstract

**Background:**

Communicating the presence of potential zoonotic pathogens such as *Plasmodium* spp. in wild animals is important for developing both animal and human health policies.

**Methods:**

The translocation of an exotic and invasive population of *Leontopithecus chrysomelas* (golden-headed lion tamarins) required the screening of these animals for specific pathogens. This studies objective was to investigate *Plasmodium* spp. infection in the *L. chrysomelas*, both to know its prevalence in these animals in the local area and to minimize the risk of pathogens being translocated to the destination site. To investigate *Plasmodium* spp. infection, blood samples from 268 animals were assessed for the presence of *Plasmodium* spp. by genus-specific PCR and stained thick and thin blood smears were examined by light microscopy. Data of human malaria infection in the studied region was also assembled from SINAN (Diseases Information System Notification—Ministry of Health of Brazil).

**Results:**

Results from the PCR and microscopy were all negative and suggested that no *L. chrysomelas* was infected with *Plasmodium* spp. Analysis of SINAN data showed that malaria transmission is present among the human population in the studied region.

**Conclusions:**

This study is the first to provide information on *Plasmodium* spp. infection in *L. chrysomelas.Plasmodium* spp. infection of this species is rare or absent though malaria parasites circulate in the region. In addition, there is minimal risk of translocating *Plasmodium* spp. infected animals to the destination site.

## Background

Malaria is a disease caused by infection with *Plasmodium* spp. parasites. It causes significant morbidity and mortality with 143,415 confirmed cases in Brazil in 2014 [1]. As well as infecting humans, *Plasmodium* spp. also infect other animals including non-human primates.

In Brazil, there are two *Plasmodium* spp. regularly identified in non-human primate populations, *Plasmodium brasilianum* and *Plasmodium simium* [2]. Differently from the majority of other primate-infective *Plasmodium*, which tend to infect hosts within the same taxonomic family, *P. brasilianum* infects hosts from at least three families of primates [3], including the family Callitrichidae [2] to which *Leontopithecus* spp. belong [4].

According to some researchers, malaria can be considered a zoonosis [5]. In the case of *P. brasilianum,* humans can be infected when exposed to sporozoites or blood stage parasites from non-human primate infections and it is transmitted by mosquito vectors to which both non-human primates and humans are exposed. *Plasmodium brasilianum* and *P. simium* are genetically and morphologically very similar to human parasites *Plasmodium malariae* and *Plasmodium vivax,* respectively [6–8]. Additionally, the human parasite *Plasmodium falciparum* has also been documented in non-human primates in the Brazilian Amazon [9]. Recently, in the Venezuelan Amazon, 12 individuals were found to be infected with parasites that had identical 18S gene sequences to *P. brasilianum* that had been isolated from the spider monkey (*Alouatta seniculus*) [10]. Therefore, it is possible that non-human primates in Brazil are reservoirs for parasites that could infect humans.

The translocation of wild animals has been highlighted as an important factor in emerging infectious diseases of wild animals [11], which in turn can threaten the survival of endangered species [11]. There is a possibility in any translocation programme that the introduction of animals into a new geographical area will involve the risk of also introducing new pathogens [12, 13], and therefore there is a responsibility to take steps to minimize this risk.

Due to the presence of non-human primate *Plasmodium* spp. infections [14] and the *Plasmodium* spp. vectors [15] in the Atlantic forest as well as locally acquired *Plasmodium* spp. infections in humans in Rio de Janeiro state [16, 17] it was determined that non-human primates living in the Atlantic forest around Rio de Janeiro could be at risk of being infected with *Plasmodium* spp. parasites.

The translocation of an exotic invasive population of *Leontopithecus chrysomelas* (golden-headed lion tamarin) [18] living in the urban Atlantic forest in Niteroi, Rio de Janeiro state to their native area (Bahia State, Brazil) [19], required the screening of the *L. chrysomelas* animals for pathogens, including *Plasmodium* spp., according to a national ruling in Brazil (IN no 179/08) [20] and International recommendations [21]. The animals were being translocated due to the risk of competition and hybridization with the endemic and endangered [22] *Leontopithecus rosalia* (golden lion tamarin).

The primary outcome of the screening was to identify whether there was any risk of translocating *Plasmodium* spp. infected animals from the original site (Rio de Janeiro state) to the destination area (Bahia state) during the *L. chrysomelas* translocation program, where they may pose a risk to both translocated animals and the local animals (although there was no golden-lion tamarin in the release site, there are other primate species). Alternatively, in cases of zoonosis, potentially presenting a risk to the human population living in the area [11, 13, 23]. In addition, information on the presence of *Plasmodium* spp. parasites in these animals could provide important information on the likelihood of these animals being reservoirs of parasites that could cause human disease. A small survey of *Plasmodium* spp. has already been conducted for *L. rosalia* with negative results (reviewed in [2]), however, this is the first work investigation involving *L. chrysomelas*.

## Methods

### Animals and capture

**Fig. 1:**
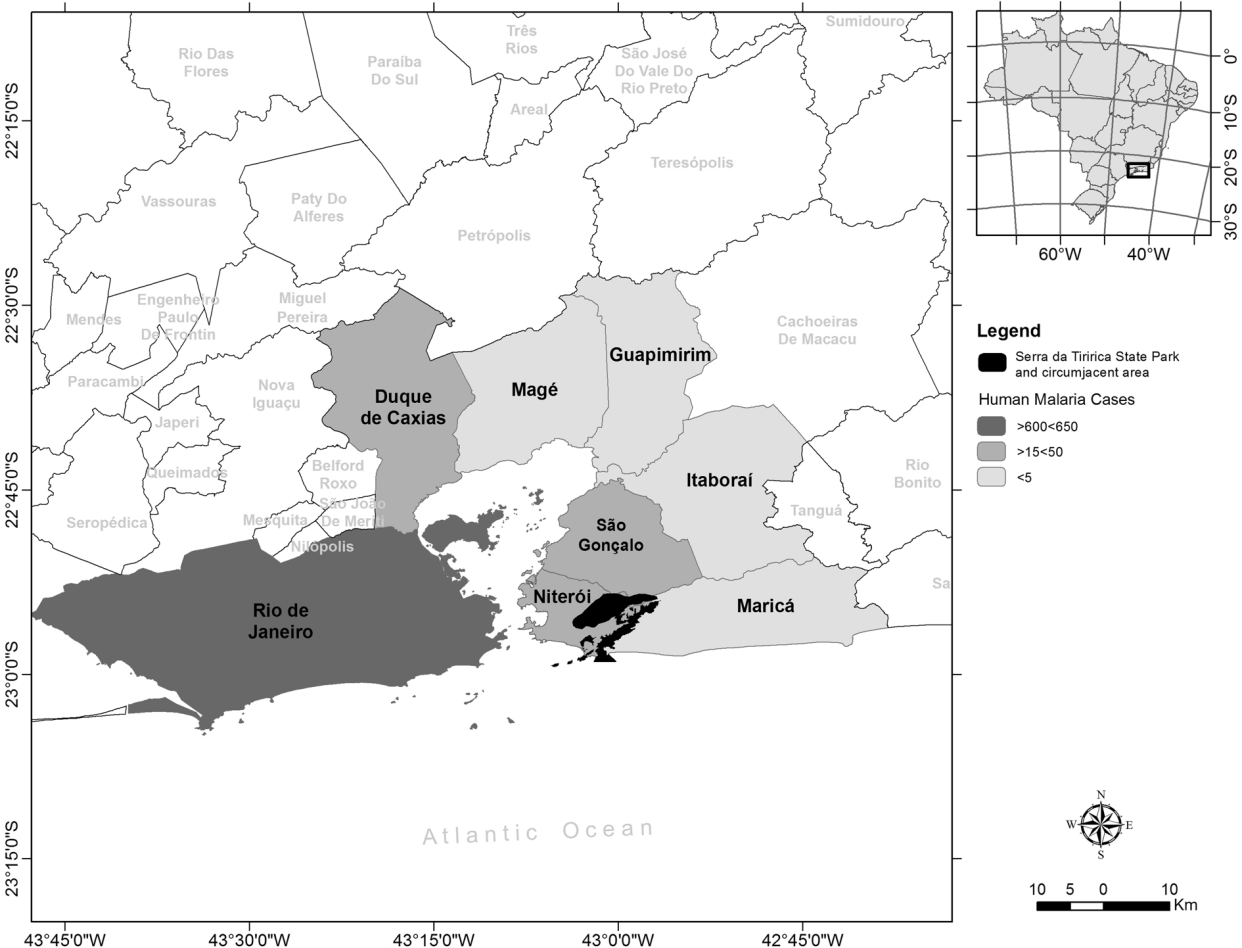
Map of Rio de Janeiro State. Serra da Tiririca State Park, where the animals were captured, is marked in *black*. The surrounding municipalities of interest are coloured in different *shades of grey* depending on the number of human malaria infections that occurred in each municipality between 2001 and 2014

Three hundred and thirty-five *Leontopithecus chrysomelas*, Family *Callitrichidae*, were captured in an urban Atlantic forest fragment in the municipality of Niterói (Serra da Tiririca State Park, 22°56′S, 43°00′W) in the state of Rio de Janeiro, Brazil (Fig. 1) between June 2012 and November 2013. Their capture was part of a programme (the Tamarins Translocation Project) to remove *L. chrysomelas* from the range of *L. rosalia* and to translocate this invasive *L. chrysomelas* population to their natural area of occurrence in another state of Brazil (Bahia). No animals suffered, died naturally or were euthanized for the purpose of, or during the course of this research.

### Quarantine, anesthesia and sample collection

After capture, animals were kept in quarantine for 30 days at the Rio de Janeiro Primatology Centre (CPRJ/INEA) in Guapimirim (22°32′S, 42°59′W) before being translocated to Bahia state. During quarantine, the animals were kept in a quiet place, away from human contact, in large and modular cages. The size of the cages varied according to the number of animals per group and dividers for visual separation between the groups were used. In each cage, there were tree trunks for the animals to move around, and wooden housing for the animals to sleep or hide in (mimicking the behaviour of the species in the wild). Protective measures were taken to prevent excessive exposure to heat including water diffusers on the ceiling of the room and air conditioners inside the building. The animals received water ad libitum and food (fruits, vegetables, chow primates, eggs, mealworms) two times a day. All measures to avoid stress and to ensure the well-being were applied and this also helped with the translocation process. To collect blood, chemical restraint was performed using 8–10 mg/kg of ketamine (CETAMIN^®^, Syntec) with 0.25 mg/kg midazolam hydrochloride (Dormium^®^, União Química) in order to conduct a clinical evaluation which included venous blood sampling and noting the animals age and sex.

### Processing and analysis of samples for *Plasmodium* spp

Thick and thin blood smears of venous blood were made and stained with Giemsa, using standard methods [24] and examined under 1000× light microscopy. One to two hundred µl of packed red blood cells were stored at −20 °C. Within 3 weeks of collection, DNA was extracted from the packed red blood cells using the Illustra™ blood genomicPrep Mini Spin kit (GE Healthcare) following the manufacturer’s instructions and genus specific nested PCR (first reaction rPLU1: 5′ tcaaagattaagccatgcaagtga 3′ and rPLU6: 5′ cgttttaactgcaacaattttaa 3′; second reaction PLU3: 5′ tttttataaggataactacggaaaagctgt 3′ and rPLU4: 5′ tacccgtcatagccatgttaggccaatacc 3′ was carried out according to previously described protocols [25, 26], in order to detect the presence of *Plasmodium* spp. DNA. Further, 10 % of samples from adults were randomly selected and re-tested by using *Plasmodium* ssrRNA primers rPLU6 5′ ttaaaattgttgcagttaaaacg 3′ and rPLU5 5′ cctgttgttgccttaaacttc 3′, as defined by Snounou et al. [27]. A blood sample from an individual known to be infected with *Plasmodium* was run as positive control in each PCR reaction. The DNA was extracted in the same way for the control as for the samples.

### Obtaining information of local infections

Data on *Plasmodium* spp. infections in humans in Rio de Janeiro state during the period 2001–2014 were obtained from the Ministry of Health of Brazil—Diseases Information System Notification (Sistema de Informação de Agravos de Notificação—SINAN)—website [28]. The area of interest was defined as Rio de Janeiro State and then within Rio de Janeiro State the municipalities around the Tiririca State Park (Rio de Janeiro, Duque de Caxias, Magé, Guapimirin, Itaboraí, São Gonçalo, Niterói, and Maricá) (Fig. 1).

### Ethics

All procedures were approved by the Ethical Principles in Animal Research of the School of Veterinary Medicine and Animal Sciences, University of São Paulo (Protocol number no 2662/2012 issued on 15/08/2012) and were in full compliance with federal permits issued by the Brazilian Ministry of the Environment (SISBIO no 30939-5 issued on 18/08/2012).

## Results

**Fig. 2:**
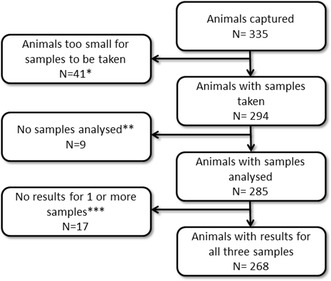
Flow chart of animals captured and tested for infection with *Plasmodium* spp. between 2012 and 2013. *Animals weighing less than 300 g were not sampled. **Nine animals not tested for *Plasmodium* as they were not to be translocated to Bahia. ***Three PCRs, 5 thin smears and 10 thick smears from 17 animals did not give results due to bad quality or lost samples

Three hundred and thirty-five animals from 56 groups were captured; most groups were between 4 and 8 animals in size. Of the 335 animals, 268 animals (127 male, 116 female, 25 of unknown sex, including 24 infants) were tested for *Plasmodium* spp. and had results for all three samples (thick and thin smears and PCR of peripheral blood); some animals were not sampled due to body size (see Fig. 2). No *Plasmodium* spp. were visible in any of the smears and none of the blood samples from the animals contained DNA that was amplified by *Plasmodium* spp. specific primers.

According to SINAN from the Brazilian Ministry of Health [28] the number of human malaria cases has increased each year in Rio de Janeiro state, with 945 cases of *Plasmodium* spp. infections reported in the state between 2001 and 2012, including 200 cases from 2012. Between 2013 and 2014, 141 new cases of *Plasmodium* spp. infection were reported in the state [28]. Moreover, reported cases from the municipalities near to the Serra da Tiririca State Park, comprise of malaria infections of residents or non-residents that were acquired elsewhere and from residents or non-residents that were acquired in the region (Fig. 1; Table 1).

**Table 1:** *Plasmodium* infections in humans^a^ between 2001 and 2014 reported in the area of interest of Rio de Janeiro State^b^, according to the—Diseases Information System Notification (SINAN) from Brazilian Ministry of Health

	*P. falciparum*	*P. vivax*	*P. falciparum* + *P. vivax*	Total
Imported cases	758	157	6	921
Autochthonous cases	8	6	1	15
Reported cases^a^	766	163	7	936

^a^Includes infections that were reported (imported and autochthonous cases) within the municipalities nearby the park, infections could occur in residents (see in Fig. 1) or non-residents

^b^The area of interest are the municipalities Rio de Janeiro, Duque de Caxias, Magé, Guapimirin, Itaboraí, São Gonçalo, Niterói, and Maricá* near the Serra da Tiririca State Park (see in Fig. 1)

## Discussion

This study describes the screening of a large wild non-human primate population (*L. chrysomelas*) for *Plasmodium* spp. None of the animals were infected. Results agree with previous work, which found *Plasmodium* spp. infection absent in other tested animals of the genus (*L. rosalia*), and only very low frequency infection of family *Callitrichidae*, even in areas where infection in other species was common [2, 9, 25, 29, 30]. For example, infection of the family *Callitrichidae* with *Plasmodium* spp. has been found in only four animals out of 604 *Callitrichidae* animals tested in Brazil between 1937 and 2013 [2, 9, 25, 29, 30] with the parasite being identified as *P. brasilianum*. In addition two species *Callithrix penicillata*, and *Callithrix jacchus* that were also introduced in Rio de Janeiro, and can be observed in the same area as the introduced *L. chrysomelas* [31], have also been surveyed for *Plasmodium* spp. infection (at other geographical locations) by light microscopy and like the *L. chrysomelas* were negative for infection [2].

Parasites do not randomly infect hosts, it is unlikely that any *Plasmodium* spp. infects all available primates, although it is still unclear which *Plasmodium* spp. can infect which species of primates. *Plasmodium brasilianum* has been shown to be able to infect a wide variety of non-human primates (reviewed in [3]), but *L. chrysomelas* may not be susceptible to infection. This could be because their position in the canopy, sleeping in hollows of trees from dusk to sunrise [32], when *Anopheles* are more active, and their small body size may result in low vector exposure (reviewed in [33]). Alternatively, *L. chrysomelas* may not provide an environment suitable for parasite survival [3]. It is also possible that the negative results are due to a low frequency of *Plasmodium* spp. in the area.

It is unlikely that this work contains false negative results as the negative results were due to agreement between both the microscopy results of thin and thick smear slides as well the as genus specific PCR, furthermore, the results were further confirmed with a different set of PCR primers in 10 % of randomly selected samples.

Though this study agrees with findings in the literature, which shows animals from the same genus or even from the same family are often negative for infection [2, 9, 25, 29, 30], it is still possible that other non-human primates in the area are infected with *Plasmodium* spp. Data from the website SINAN showed that endemic infections in areas around the park do occur (Table 1) and interestingly *Anopheles aqualasis,* an efficient transmitter of *Plasmodium* spp., has been identified specifically in the same area where the *L. chrysomelas* were captured [34]. In addition, other studies in the Atlantic forest have reported *Plasmodium* spp. infections in vectors [15] and non-human primates [14], including in 30 % of tested non-human primates living in and around the Rio de Janeiro Primatology Center (CPRJ/INEA) in Guapimirim [35] which is located the same area of this study.

*Leontopithecus chrysomelas* in Rio de Janeiro often live in high-risk interfaces, in close proximity with humans occasionally accessing backyards or housing, which could increase the risk of transmission, especially when human residents are infected with *Plasmodium*. In addition, if they were infected with *Plasmodium*, this proximity would identify them as possible reservoirs for *Plasmodium* spp. which may in turn infect the local population; however these results suggest that this is unlikely to occur.

With the translocation of the animals, there was the responsibility to ensure that this action would not also result in the transfer of pathogens to potentially susceptible wild animals and/or the local human population. The results from this work show that there is a minimum risk of transfer of *Plasmodium* spp. to Bahia state. However, establishing the risk factors for emerging infectious diseases is challenging and even the introduction of disease-free animals into an area could pose a threat as it has the potential to alter variables like population density [11], therefore, any translocation programme needs to be thoroughly thought through.

Much research still needs to be done in the field of *Plasmodium* in non-human primates. The evolutionary history of *P. brasilianum* and *P. simium* is unclear (primarily due to a lack of primate samples [7]). However, data suggests that the transfer from humans to non-human primates (or vice versa) was recent and in the case of *P. brasilianum* has occurred more than once [7]. Also, though *P. brasilianum* can infect humans in an experimental setting [5], it is still unclear if it is likely to pose a risk to humans. As many countries strive to eliminate malaria, whether species such as *P. brasilianum* and *P. simium* can be readily transmitted between non-human primates to human populations needs to be clarified. In addition, any animal population likely to be a reservoir needs to be identified.

## Conclusions

This study is the first to provide information about *Plasmodium* spp. infection in a population of *L. chrysomelas* of the urban Atlantic forest of Rio de Janeiro state. The results indicate that *Plasmodium* spp. infection in *L. chrysomelas* population, from Serra da Tiririca Park, is rare or absent despite malaria parasites circulating in the region among vectors, humans and other non-human primates. The absence of infection provides no evidence to support that this species is a reservoir for parasites infecting humans and also suggests that the translocation of the tested animals exhibits a minimal risk of translocating *Plasmodium* spp. infected animals to Bahia state. In addition, this study promotes knowledge about *Plasmodium* parasites in the same areas that endangered species inhabit, such as *Leontopithecus rosalia*, which could represent a further threat for these tamarins and other primates.

## Abbreviation

SINAN: Sistema de Informação de Agravos de Notificação website
